# Supplementary material for: mRNA Vaccine Technology Beyond COVID-19
Source: Vaccines (Basel). 2025 May 31;13(6):601. doi: 10.3390/vaccines13060601 (PMC12197385; doi:10.3390/vaccines13060601)
Supplement: Supplementary file 1 [file vaccines-13-00601-s001.zip › vaccines-3640680-supplementary.pdf]

Table 2: List of ongoing clinical trials involving mRNA therapies for cancers

Company	Candidate	Product Type	Cancer Type	Status	CT Ref
BioNTech	BNT111 TYR, vaccine, CTAG1B	mRNA vaccine	Malignant melanoma	Phase II	NCT04526899
	BNT113 vaccine	mRNA vaccine	Head and neck cancer	Phase II	NCT04534205
BioNTech/Regeneron Pharmaceuticals	BNT116 vaccine, TAA	mRNA vaccine	Non-small cell lung cancer (NSCLC)	Phase II	NCT05557591
BioNTech/Roche	BNT112 (autogene cevumeran) vaccine, neoantigen	mRNA vaccine	Malignant melanoma	Phase II	NCT03815058
			Colorectal cancer	Phase II	NCT04486378
			Other metastatic tumors	Phase II	NCT03289962
			Pancreatic ductal adenocarcinoma (PDAC)	Phase II	NCT05968326
			Muscle invasive urothelial carcinoma (MIUC)	Phase II	NCT06534983
		****	Metastatic castration resistance prostate cancer (mCRPC)	Phase I/II	NCT04382898
Moderna TX, Inc.	mRNA-4359 + pembrolizumab		Advanced solid tumors	Phase I/II	NCT05533697
	mRNA-4106 + Nivolumab/Relatlimab		Solid tumors	Phase I	NCT06880549
	mRNA-2752		Refractory solid tumors/lymphoma	Phase I	NCT03739931
	mRNA-2736		Relapsed/refractory multiple myeolema	Phase I	NCT05918250
Moderna/ Merck & Co	mRNA-4157 vaccine/ neoantigen	mRNA vaccine	Bladder cancer	Phase I/II	NCT06305767
			non-small cell lung cancer (NSCLC)	Phase III	NCT06077760
			Renal cell carcinoma	Phase II	NCT06307431
			Malignant melanoma	Phase III	NCT05933577
			Others	Phase II/III	NCT06295809
Stemirna		mRNA vaccine	Advanced malignant	Preclinical	NCT05949775

			solid tumors		
			Personalized tumor vaccine for esophageal and non-small cells lung cancers	Preclinical	NCT03908671
CureVac	CV09050101	mRNA vaccine (CVGBM)	Glioblastoma (GBM)	Phase I	NCT05938387
Fudan University	PANC-IIT-RGL-mRNA vaccine	mRNA vaccine	Pancreatic adenocarcinoma	Preclinical /phase I	NCT06156267
	HRXG-K-1939	mRNA vaccine + adebrelimab	Advanced solid tumors	Phase I	NCT05942378
Guangzhou Medical University	ZZVACCINE-mRNA-020	Neoantigen mRNA vaccine	Advanced Solid Tumors	Phase I	NCT06195384
National cancer institute ***	NCI-4650	mRNA-based personalized vaccine	Metastatic epithelial cancer	Phase I/II	NCT03480152
Ruijin Hospital	Xp-004	mRNA tumor vaccine	Recurrent pancreatic cancer	Phase I	NCT06496373
	WGc-043	EBV mRNA vaccine	Refractory Lymphoma	Phase I	NCT06788600
Guangdong Provincial People's Hospital		Neoantigen mRNA vaccine	non-small cell lung cancer (NSCLC).	Phase I	NCT06735508
University Hospital Tuebingen	RNA-Mel-03	mRNA vaccine coding for Melan-A, Mage-A1, Mage-A3, Survivin, GP100 and Tyrosinase	Malignant melanoma	Phase I/II	NCT00204516
Shanghai Zhongshan Hospital		Personalized mRNA vaccine	Postoperative hepatocellular carcinoma	Preclinical	NCT05761717
Steinar Aamdal***	DC-004	mRNA vaccine with dendritic cells	Metastatic malignant melanoma	Phase I/II	NCT00961844

Radboud University Medical Center		TLR-DC and Trimix DC loaded with mRNA encoding melanoma-associated tumor antigens (gp100 and tyrosinase)	Metastatic melanoma	Phase I/II	NCT01530698
*** Memorial Sloan Kettering Cancer Center		Vaccination With CT7, MAGE-A3, and WT1 mRNA-electroporated Autologous Langerhans-type Dendritic Cells as Consolidation	Multiple myeloma	Phase I	NCT01995708
		Autologous Langerhans-type Dendritic Cells with mRNA encoding tumor antigen	Melanoma	Phase I	NCT01456104
Shanghai Jiao Tong University School of Medicine	mRNA-0523-L001	Individualized mRNA neoantigen vaccine	Advanced endocrine tumor	Phase I	NCT06141369
John Sampson, Duke University	BTSC mRNA-loaded DCs	Personalized cancer vaccine	glioblastoma multiforme (GBM)	Phase I	NCT00890032
Sir Run Run Shaw Hospital	iNeo-Vac-R01	Personalized mRNA vaccine	Advanced Digestive System Neoplasms	Phase I	NCT06019702
		iNeo-Vac-R01 + standard adjuvant therapy			NCT06026774
Inge Marie Svane, Herlev		mRNA transfected	Metastatic prostate cancer	Phase II	NCT01446731

Hospital		dendritic cell			
Steinar Aamdal, Oslo University Hospital ***	DC-004 **	mRNA vaccine therapy with dendritic cells	Metastatic malignant melanoma	Phase I/II	NCT00961844
	DC-006 **	Dendric cells with amplified ovarian cancer stem cell vaccine	Recurrent Platinum Sensitive Epithelial Ovarian cancer	Phase I/II	NCT01334047
	DC-005	Dendritic cells with cancer mRNA vaccine	Prostate cancer	Phase I/II	NCT01197625
	DC-CAST-GMB	Dendritic cells with cancer mRNA vaccine	Globalstoma	Phase I/II	NCT00846456
Nanjing Tianyinshan Hospital	RGL-270	MRNA Vaccine + Adebrelimab	Non-small cells lung cancer	Phase I	NCT06685653
Radboud University Medical Center	TLR-DC and Trimix DC	Autologous dendritic cell vaccine	Melanoma	Phase I/II	NCT01530698
		mRNA vaccine	Melanoma stage III/IV	Phase I/II	NCT00243529
		MiHA-loaded PD-L-silenced DC vaccine	Hematological malignancies	Phase I/II	NCT02528682
		CEA-loaded dendritic cell vaccine	Colorectal cancer	Phase I/II	NCT00228189
***Ludwig Institute for Cancer Research	BI 1361849	mRNA vaccine + durvalumab + tremelimumab	Metastatic non-small cells lung cancer	Phase I/II	NCT03164772
YueJuan Cheng, Peking Union Medical College Hospital	KY- 1007	Personalized mRNA neoantigen vaccine	Advanced solid tumors	Early phase I	NCT05359354
Guangdong 999 Brain Hospital	PERCELLVAC3	Personalized cellular vaccine	Glioblastoma	Phase I	NCT02808416
	PerCellVac2	Personalized cellular vaccine	Glioblastoma	Phase I	NCT02808364
Jinling Hospital,	SJ-Neo006	Camrelizumab + personalized	Pancreatic cancer	Early phase I	NCT06326736

China		neoantigen vaccines			
Wu Wenming, Peking Union Medical College Hospital	XH001	Neoantigen cancer vaccine + Ipilimumab	Pancreatic cancer	Early phase I	NCT06353646
University of Florida ***	RNA PRIME	pp65 RNA LP (DP1 & DP2) vaccines	Pediatric Recurrent Intracranial malignancies	Phase I/II	NCT05660408
	pp65 RNA-LP	mRNA vaccine	Recurrent glioblastoma	Phase I	NCT06389591
	PNOC020	RNA-loaded lipid particle (RNA-LP) vaccine	Pediatric High-Grade Gliomas (pHGG), and Adult Glioblastoma	Phase I/II	NCT04573140
	pp65-shLAMP ***	pp65-shLAMP mRNA DCs with GM-CSF	Glioblastoma Multiforme	Phase II	NCT02465268
jianming xu, The Affiliated Hospital of the Chinese Academy of Military Medical Sciences		mRNA neoantigen tumor vaccine	Advanced gastric, esophageal, and liver cancers	Early phase I	NCT05192460
	XH001	Neoantigen tumor vaccine, XH001 + sintilimab	Advanced solid tumors	Preclinical	NCT05940181
RinuaGene Biotechnology Co., Ltd.	RG002	mRNA therapeutic vaccine	HPV16/18 associated Cervical Intraepithelial neoplasia	Phase I/II	NCT06273553
Gary Archer Ph.D., Duke University		Dendric cell-based mRNA vaccine	Brain tumor	Phase I	NCT00639639
		DC-based mRNA vaccine + Nivolumab	Brain tumors	Phase I	NCT02529072
University Medical Center Groningen	W_ova1 vaccine	Liposome formulated mRNA vaccine	Ovarian cancer	Phase I	NCT04163094
Zwi Berneman, Unive rsity Hospital,	CCRG12-001	DC vaccine	Acute myeloid leukemia (AML)	Phase II	NCT01686334

Antwerp					
Svein Dueland, Oslo University Hospital		mRNA vaccine	Malignant melanoma	Phase I/II	NCT01278940
		mRNA vaccine	Androgen Resistant Metastatic Prostate cancer	Phase I/II	NCT01278914
Inge Marie Svane, Herlev Hospital		DC vaccine	Breast cancer	Phase I	NCT00978913
Peking Union Medical College Hospital	ABOR2014(IPM511)	Neoantigen mRNA vaccine	Advanced hepatocellular carcinoma	Preclinical	NCT05981066
Diakonos Oncology Cooperation	DOC1021	Dendric cell-based mRNA vaccine loaded with tumor lysate	Adult Glioblastoma	Phase II	NCT06805305
Peking University Hospital & Institute	JCXH-212	Tumor neoantigen mRNA vaccine	Malignant solid tumors	Early phase I	NCT05579275
*** Merck Sharp & Dohme LLC	mRNA 5679/V941	mRNA vaccine	Metastatic Non-Small Cell Lung Cancer, Colorectal or Pancreatic Adenocarcinoma	Phase I	NCT03948763
University Hospital, Antwerp	CCRG 05-001	Dendritic-based mRNA vaccine	Acute Myeloid Leukemia	Phase I	NCT00834002
		DC vaccine	Pediatric High Grade Gliomas, and Diffuse Intrinsic Pontine Gliomas	Phase I/II	NCT04911621
The Affiliated Hospital Of Guizhou Medical University	InnoPCV	mRNA vaccine	Advanced solid tumors	Early phase I	NCT06497010
